# Quantification of sources and potential risks of cadmium, chromium, lead, mercury and arsenic in agricultural soils in a rapidly urbanizing region of southwest China: the case of Chengdu

**DOI:** 10.3389/fpubh.2024.1400921

**Published:** 2024-05-30

**Authors:** Chengyi Huang, Zhangyong Gou, Xinpeng Ma, Guitang Liao, Ouping Deng, Yuangxiang Yang

**Affiliations:** ^1^College of Water Conservancy and Hydropower Engineering, Sichuan Agricultural University, Yaan, China; ^2^College of Environmental Science, Sichuan Agricultural University, Chengdu, China; ^3^College of Resources and Environment, Chengdu University of Information Technology, Chengdu, China; ^4^College of Resources, Sichuan Agricultural University, Chengdu, China

**Keywords:** agricultural land, heavy metals, pollution evaluation, APCS-MLR receptor model, human health risks

## Abstract

Rapid urbanization a major factor affecting heavy metal contamination on suburban agricultural soils. In order to assess the dynamic contamination of heavy metals in soil from agricultural land bordering a rapidly urbanizing area and the transfer of human health risks from contaminants in this process, 186 and 293 soil samples from agricultural land in suburban Chengdu were collected in September 2008 and September 2017, respectively. Several indicators, such as the integrated pollution index (PI) and the potential ecological risk index (RI), were employed for analyzing the heavy metal contamination levels, and the APCS-MLR receptor model were applied for analyzing the heavy metal sources. As a result, mean concentrations for five elements did not exceed the national soil pollution risk screening values in the two periods mentioned above. Nemerow’s composite contamination index revealed an increase in soil contamination of arable land after 10 years of urbanization, with 3.75 and 1.02% of light and moderate sample plots, respectively, by 2017. The assessment for potential ecological risk indicated an increased level of eco-risk to high for most of the sample plots. Based on the APCS-MLR model, the origin and contribution to the five elements varied considerably between the two periods mentioned above. Among them, soil Pb changed from “industrial source” to “transportation source,” soil Cr changed from “natural source” to “transportation source,” and As and Hg changed from “industrial source” to “transportation source.” As and Hg were associated with agricultural activities in both periods, and Cd was derived from industrial activities in both periods. The study suggests that inhalation has become a major contributor to non-cancer health risks in urbanization, unlike intake routes in previous periods, and that the increase in cancer risk is mainly due to children’s consumption of agricultural products with As residues. The change in the main source of As to “transportation” also indicates a decrease in air quality during urbanization and the development of the transportation industry. This study provides a reference for the governments of rapidly urbanizing cities to formulate relevant highway and agricultural policies to safeguard the health of the people based on the current situation.

## Highlights

- Urbanization activities have the greatest impact on the accumulation of Cd in soil.
- Inhalation replaces ingestion as the main pathway for human health hazards during urbanization.
- Urbanization leads to changes in the sources of Cr in soils.

## 1 Introduction

As the economic and social development is rapid, environmental qualities in soils are affected with superposition by various parties, which brings great burden to soil environmental protection and soil health, especially heavy metal contamination in farmland has caused wide concern all over the world (1). In ecosystems, heavy metal pollution disrupts the ecological balance of the soil, inhibits microbial activity and plant growth in the soil, leading to plant withering, soil erosion and loss of biodiversity, posing a serious threat for healthy city and countryside habitats as well as for eco-safety on farmland. Based on the announcement by MOEC and MNR in 2014, China’s arable land area exceeded the standard by 19.4% (2). By contrast, western China has entered a period of accelerated urbanization since 2005, and its urbanization rate has begun to exceed that of the eastern region (3), with Chengdu rapidly growing to be one of the central cities leading the rapid economic growth and absorbing new urban population in western China, for the human body, the hazards of heavy metal pollution are also very serious. For example, lead pollution may affect the nervous system, kidneys and hematopoietic function; cadmium pollution mainly affects the liver, nervous system and may induce high blood pressure. Therefore, it is important to focus on the extent of heavy metal contamination in the agricultural lands of rapidly urbanizing regions, it also analyzes the dynamics of heavy metal pollution sources to facilitate the development of regional soil environmental protection, rational layout of agricultural production and food safety.

In the past, studies on heavy metals have mainly concentrated on content characteristics and space distribution (3), contamination levels (4). To analysis the heavy metal contamination characteristics and ecological risk of cultivated land, there are: contamination index (5), land accumulation index (6), Nemero composite contamination index (7), enrichment factor (8) and potential ecological risk index (9), etc. The US EPA-recommended health risk assessment model for soil heavy metals is commonly used in healthy impact assessments. The current pollution source analysis methods are mainly divided into two categories, i.e., qualitative source identification and quantitative source analysis. Source identification methods mainly include geostatistical analysis (10), multivariate statistical analysis (11), soil morphological classification and soil profile method, and the commonly used methods for source analysis of soil heavy metal pollution are: APCS-MLR, positive definite matrix decomposition (PMF), chemical mass balance method (CMB), UNMIX receptor model and other methods (12–14). Since Thurston and Spengler introduced the concept of absolute principal component score (APCS), combining PCA (principal component analysis) and MLR (multiple linear regression), it has been developed and extensively applied to study source analysis type of problems (15). However, the results of the source analysis of this receptor model lack visualization, therefore, combining it with geostatistics to represent the contribution of each source by geostatistical interpolation can better identify the sources, which is a new source heavy metal analysis method with simple and effective operation.

Chengdu is the most typical fast-growing city in China and the central city in the west, and is also the hub of science and technology, commerce and trade, financial center, transportation and communication in the southwest region as determined by the State Council, and is known as the “Land of Heaven.” At the same time, as an important food and agriculture production base in China, the region is also exposed to ecological risks of heavy metals in soil. Several studies have been conducted to assess the heavy metal contamination of agricultural soils in Chengdu (16, 17). The scope of studies on heavy metals in agricultural soils in the region is small, and there are few studies on the overall contamination status of heavy metals in agricultural soils in the study area, especially the quantitative changes in the accumulation of heavy metal pollutants and their sources specifically for the period of rapid urbanization development in the region. Therefore, this research combines pollution index, potential ecological risk index, human health risk evaluation, geostatistical analysis and APCS-MIR receptor model to dynamically investigate level, spatial distribution and ecological risk of heavy metal pollution in arable soils during the period of rapid urban development in Chengdu, and to quantify changes in potential sources of heavy metals in arable soils. The results of the study will have strong practical and demonstrative implications for controlling and managing farmland contamination and human health in the context of rapidly urbanizing China.

## 2 Materials and methods

### 2.1 Study area

**Figure 1:**
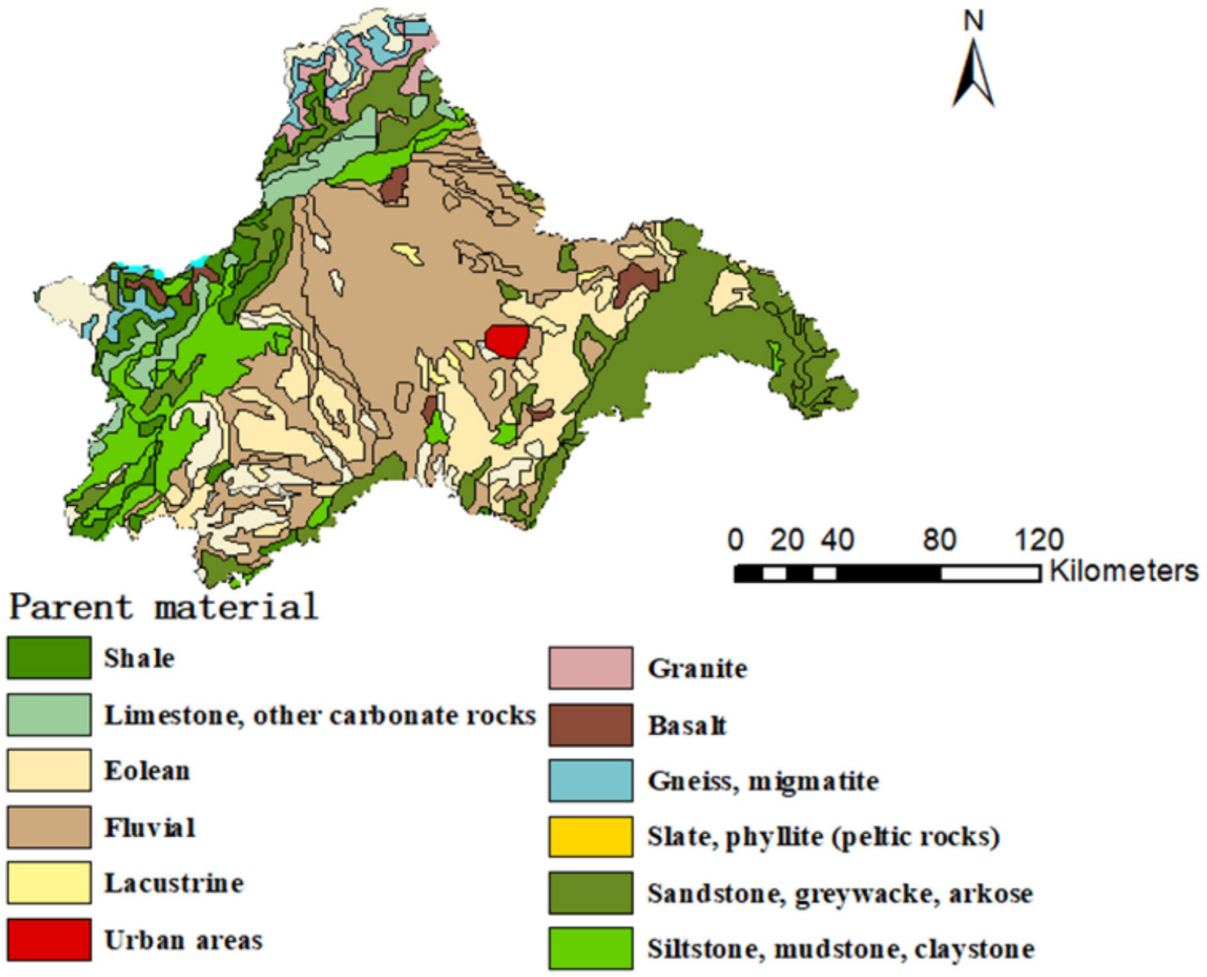
Parent material types in the study area.

Chengdu is an important central city in western China and ranks as one of the top new tier cities in China. The area is located in the hinterland of Chengdu Plain, mainly formed by the alluvium of Minjiang River, Tuo River and its tributaries and some gully flow flood deposits. The soil type is mainly rice soil and purple soil, and the soil pH range is 3.91 ~ 7.89. The area is dominated by subtropical humid monsoon climate, with annual average temperature and precipitation of 15.2–16.5°C and 900–1,300 mm, respectively, and is the main production base of agricultural products such as grain and vegetables in Sichuan basin (18). Thus, there is a need to pay greater concern to the problem of heavy metal contamination in arable land (Figure 1).

### 2.2 Sample collection and analysis

**Figure 2:**
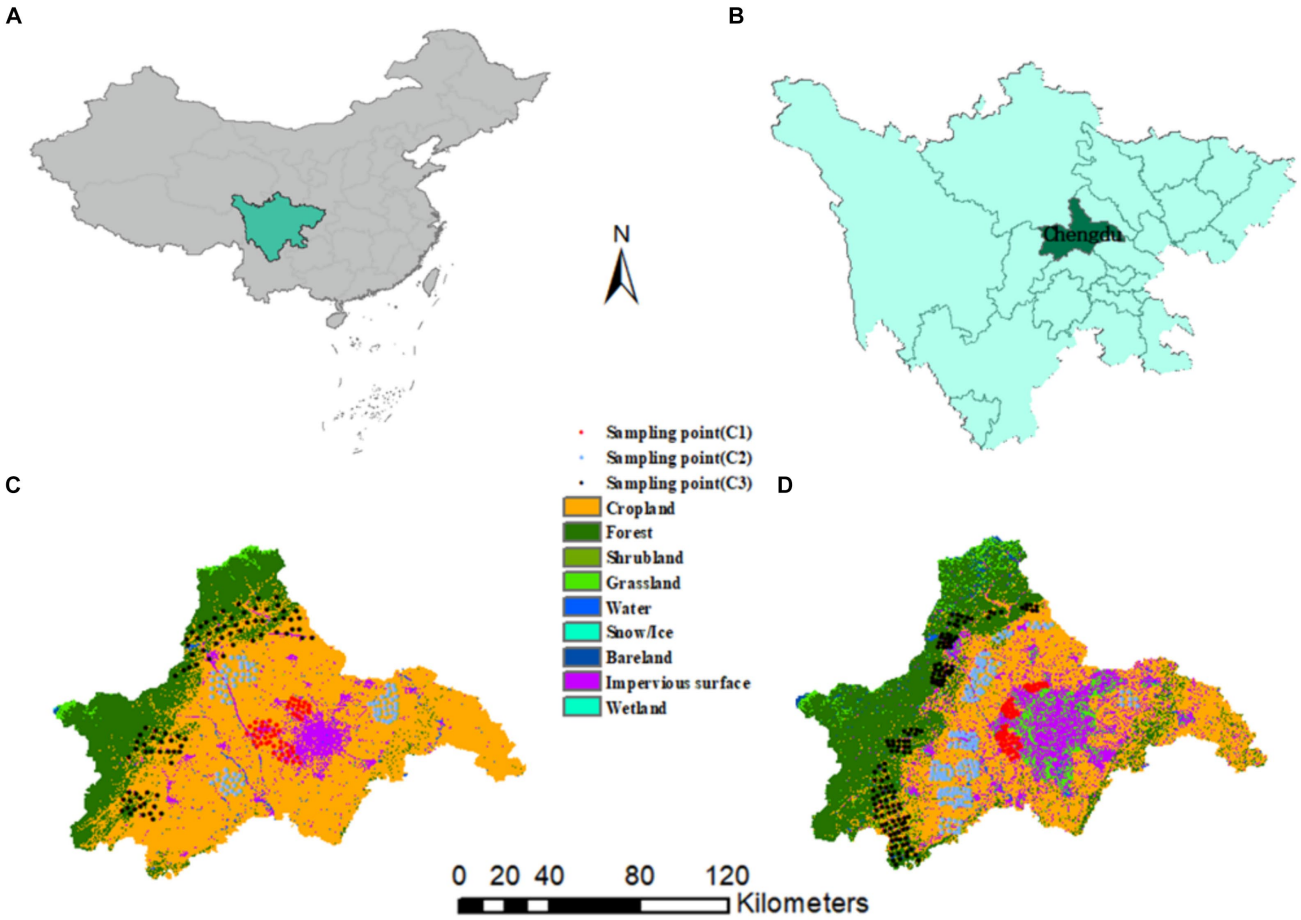
**(A,B)** geographical location of the study area, **(C)** land cover in 2008, **(D)** land cover in 2017.

Soil samples were collected in two periods, and were divided into three major circles: suburban (C1), suburban (C2), and distant suburban (C3), taking into account the economic development plan, location, land use type, and soil type of Chengdu, as shown in Figure 2. The suburban circle includes: Wenjiang district, Shuangliu county, Pixian county, Longquanyi; the suburban circle includes: Qingbaijiang, Xindu; the distant circle includes Chongzhou City, Dujiangyan City, Dayi County, etc. In September 2008, 226 soil samples were collected, with a density of about 1 sample per km^2^; after nearly 10 years of rapid development, the sampling points were displaced or increased or decreased on the basis of the original monitoring points, taking into account changes in land use types and other factors. Soil samples were collected in September 2017 with 389 samples. The coordinates of the sample points were located with a handheld GPS from Garmin, United States, and the surface soil of 0–10 cm of the cultivated layer was collected with a wooden shovel and collected according to the plum-shaped sampling method, and the soil samples were thoroughly mixed and 1.5 kg of soil samples were retained according to the quadrat method. The samples were spread out indoors to dry naturally, and foreign objects such as stones and plant roots were removed and ground and passed through a 200 mesh nylon mesh sieve for analysis and testing. Soil samples were digested by microwave digestion with HCl + HNO_3_ + HF + H_2_O_2_; As and Hg were determined by atomic fluorescence spectrometry (AFS); Cr and Pb were determined by flame atomic absorption spectrometry (AAS/FAAS); and Cd was determined by graphite furnace atomic absorption spectrophotometry (GF-AAS).

### 2.3 Evaluation methods for heavy metals

#### 2.3.1 Pollution indexes and the Nemerow integrated pollution index

Using the Soil Environmental Quality Standard (GB15618-2018) as the reference value, the five heavy metals in the soil were evaluated using the single-factor pollution index method and Nemero. The single-factor pollution index method is to analyze and assess the level of accumulated contamination of a particular heavy metal in the soil. Its calculation formula is

(1) $$P_{i}=C_{i}/S_{i}$$

Where: P*_i_* is the calculated single heavy metal pollution index; *C_i_* is the measured value of the heavy metal; S_i_ is the evaluation standard value, and the background soil value of Chengdu City is used as the reference value. P_i_ ≤ 0.7 is clean, 0.7 < P_i_ ≤ 1.0 is safe, 1.0 < P_i_ ≤ 2.0 is light pollution, 2.0 < P_i_ ≤ 3.0 is moderate pollution, and P_i_ > 3.0 is heavy pollution.

The Nemero integrated index is a combined evaluation on the cumulative pollution level of several heavy metals in soil, emphasizing the influence of highly concentrated pollutants to soil circumstance qualitative. Its calculation formula is.

(2) $$P_{N}=\sqrt{\frac{P_{\max}^{2}+P_{mean}^{2}}{2}}$$

Where: P*_N_* is the composite pollution index; P*_mean_* is the average of each individual pollution index P*_i_*; P*_max_* is the maximum of all individual pollution indexes. P*_N_* ≤ 0.7 is clean, 0.7 < P*_N_* ≤ 1.0 is safe, 1.0 < P*_N_* ≤ 2.0 is light pollution, 2.0 < P*_N_* ≤ 3.0 is medium pollution, and *P_N_* > 3.0 is heavy pollution (19).

#### 2.3.2 Potential ecological risk assessment

To further assess the ecological risk in the arable soil environment across the research region, the heavy metal toxicity response index developed by Hakanson (20) and the national soil pollution risk screening values were used as criteria to calculate the $E_{r}^{I}$ and RI values. The potential ecological risk index is used for the assessment of the extent of metal contamination in land and its potential ecological risk. It could be obtained by the formula below.

(3) $$C_{f}^{i}=C_{s}^{i}/C_{n}^{i}$$

(4) $$E_{r}^{i}=T_{r}^{i}\cdot C_{f}^{i}$$

(5) $$RI=\overset{m}{\underset{i=1}{\sum }}E_{r}^{i}$$

Where: $C_{f}^{i}$ is the pollution index of heavy metal i, $C_{s}^{i}$ and $C_{n}^{i}$ are the actual and standard values of heavy metal *i*, respectively; $E_{r}^{i}$ is the potential ecological risk index of heavy metal *i*, $T_{r}^{i}$ is the toxicity index. Hakanson gives the heavy metal toxicity index as Hg = 40 > Cd = 30 > As = 10 > Pb = 5 > Cr = 2; *RI* is the integrated potential ecological risk index. C is soil heavy metal concentration (mg/kg; Table 1).

**Table 1:** The classification of indexes.

$E_{r}^{i}$ Category	Description	RI Category	Description
≤30	Low risk	≤60	Low risk
30–60	Moderate risk	60–120	Moderate risk
60–120	High risk	120–240	High risk
>120	Extreme risk	≥240	Extreme risk

#### 2.3.3 Human health risk evaluation

The research use the method recommended by the U.S. Environmental Protection Agency to evaluate the health risks of five heavy metals in cropland soils. The research community was classified to two categories: adults and children, based on behavioral and physiological differences. The model assumes that heavy metals enter the body primarily through different pathways. Cancer-causing and non-cancer-causing heavy metals were considered below (21).

(6) $$ADI_{ing}=C\times \frac{lngR\times EF\times ED}{BW\times AT}\times 10^{-6}\;$$

(7) $$ADI_{inh}=C\times \frac{lnhR\times EF\times ED}{PEF\times BW\times AT}$$

(8) $$ADI_{derm}=C\times \frac{SA\times AF\times ABS\times EF\times ED}{BW\times AT}\times 10^{-6}$$

Where: AD_Iing_ is intake, AD_Iinh_ is inhalation and ADI_derm_ is average daily exposure to heavy metals from dermal exposure; C is soil heavy metal concentration (mg/kg). Other notes are as in the text (22). In this study, the carcinogenic risk and non-carcinogenicity of soil heavy metals were calculated as the following formulas:

(9) $$HQ_{i}=ADI_{i}/RfD_{i}$$

(10) $$HI=\sum HQ_{i}$$

(11) $$CR_{i}=ADI_{i}/SF_{i}$$

(12) $$TCR=\sum CR_{i}$$

If CR/TCR is between 10^−6^ and 10^−4^ indicates acceptable risk, if CR/TCR If the CR/TCR is between 10 and 10^–6-4^, the risk is acceptable, if the CR/TCR is greater than 10, there is a significant health risk of cancer, and if the CR/TCR is less than 10, there is no health hazard (21).

#### 2.3.4 Classification of coefficient of variation

The Classification of coefficient of variation (CV) is an indicator to evaluate the level of divergence of the probability distribution, and in general, the greater the divergence, the greater the coefficient of variation. CV according to related studies (23). If the CV is less than 0.16 the element is in mild variability, if the CV is greater than 0.16 less than 1 it is in moderate variability, and if the CV is greater than 1 it is in high variability.

### 2.4 APCS-MLR receptor model

Factor analysis was applied to resolve the content of individual heavy metals in the research region in order to clarify the sources of heavy metals in the land. Factor analysis is a method of attributing variables with complex associations to a representative number of independent factors, yielding results that retain more of the original information (24). The model assumes a linear relationship between the content of soil or atmospheric pollutants and the contribution of pollution sources, converts the j results of factor analysis into absolute principal factor scores (APCS), and then performs a multiple linear regression of each heavy metal content on all APCSs separately. The regression coefficients were considered as key indicators for the quantitative analysis of pollutant source contributions.

Assume a sample whose concentration is 0, and then calculate its factor score as follows.

(13) $$Z_{i0}=\frac{0-{\bar{C}}_{i}}{\sigma_{i}}$$

APCS was obtained by subtracting the 0-sample score from each actual score, and finally a multiple linear regression was conducted with APCS as the independent variable and heavy metal content as the dependent variable, and the regression coefficients and APCS were transformed into the contribution of pollutant sources by the formula.

(14) $$C_{i}=b_{i0}+\overset{p}{\underset{i=1}{\sum }}\left(b_{pi}\times APCS_{p}\right)$$

Where: b_pi_ is the regression coefficient and APCS_p_ is the absolute factor score of factor p (25).

### 2.5 Analysis of experimental data

In this study, statistical analyses, normality tests and Person correlation analyses were performed for the five heavy metals with SPSS 22.0 software. Correlation analyses for the APCS-MLL receptor model were done with Excel.

## 3 Results and discussion

### 3.1 Description statistical of heavy mental concentrations

Description statistics were analyzed for five heavy metals in the Chengdu farm lands (Table 2). Compared with 2008, mean content for Cd, As, and Cr increased from 0.18, 6.91, and 42.25 mg/kg^−1^ to 0.30, 9.21, and 88.29 mg/kg^−1^ in 2017, with increases of 66.67, 33.28, and 108.97%, respectively, while the average contents of soil Pb and Hg increased from 43.04 mg/kg^−1^ and 0.09 mg/kg^−1^ to 40.46 mg/kg^−1^ and 0.06 mg/kg^−1^ with a decrease of 5.99 and 33.33%, respectively. Except for soil Cr and Hg in 2008 and Hg in 2017, the averaged content in both phases was above the background value of soil elements in Chengdu city and compared with the national soil pollution risk screening value, the averaged content in both phases was below the national risk screening value.

**Table 2:** Statistical analysis of heavy metal content of soil in the study area.

Element	Year	Maximum value	Minimum value	Average value	Standard deviation	Coefficient of variation	Kurtosis	Skewness	Soil background values	Screening value
Cd	2008	0.52	0.05	0.18	0.06	0.329	5.428	1.115	0.172	0.5
	2017	0.90	0.05	0.30	0.13	0.424	3.307	1.216		
Pb	2008	101.04	19.40	43.04	10.32	0.240	6.952	1.501	23.1	80
	2017	299.13	14.61	40.46	32.82	0.811	34.037	5.420		
As	2008	27.34	0.34	6.91	2.59	0.374	20.726	2.803	6.42	25
	2017	20.24	0.00	9.21	3.94	0.428	0.176	0.093		
Cr	2008	551.96	16.70	42.25	40.44	0.957	138.02	10.988	70.3	300
	2017	189.00	47.80	88.29	23.66	0.268	4.541	1.950		
Hg	2008	1.00	0.00	0.09	0.12	1.428	16.660	3.128	0.181	0.5
	2017	0.89	0.00	0.06	0.09	1.572	40.138	5.230		

“-” indicates that the heavy metal was not detected in the soil sample; except for kurtosis, skewness and coefficient of variation, the units are mg/kg^−1^.

The CV of soil Cd, Pb, As, and Cr were medium to high variability (0.16 < CV < 1) in both periods, and the highest coefficients of variation of Hg content were 1.428 and 1.572 reaching strong variability levels (CV > 1) in both periods, respectively. Overall, the five heavy metals belonged to medium and high level of variations in these two periods, while the variation coefficients of the other four heavy metals, except for soil Cr, increased after the rapid urban development in the last decade, showing that the variations of heavy metals in agricultural soils in the whole research region were high, spatially dispersed and subject to higher possibility of anthropogenic influence.

### 3.2 Heavy metal pollution assessment and risk assessment

Based on the evaluation criteria of the five heavy metals P_i_ and P_N_ (Equations 1,2), the percentage of contamination levels of each heavy metal was analyzed (Table 3). As seen in Pi, the majority of sample sites for the five heavy metals were uncontaminated in both periods. But in this study period, the contamination level of soil As at all monitoring sample points was always non-polluting, and the proportion of light contamination of soil Hg decreased, while the contamination levels for the other three heavy metals increased, with soil Pb being the worst, whose proportion of light, moderate and heavy contamination sample points increased from 1.08, 0 and 0% in 2008 to 2.5%. 1.08, 0 and 0% in 2008 to 2.05, 1.37 and 1.02%. And it can be seen by the P_N_ that the sample points showing mild pollution in the arable land of the research region showed an increase from 1.08 to 3.75%, 2017, and 1.02% of sample points with moderate pollution level appeared in 2017. It indicates that during the rapid urbanization process in Chengdu city in the last decade, some of the farmland soils have been subjected to more serious anthropogenic impacts.

**Table 3:** Heavy metal pollution index and ecological risk index of cultivated soils in the study area.

Element	Year	Pollution index	Proportion of sample sites with different pollution levels (%)				Ecological hazard index	Proportion of sample sites with different ecological risk levels (%)			
			Non-polluting	Mild	Moderate	Severe		Minor	Moderate	Strong	Very strong
Cd	2008	P_i_	99.46	0.54	0.00	0.00	$E_{r}^{i}$	39.25	59.68	1.08	0.00
	2017		93.52	6.48	0.00	0.00		11.95	58.70	28.33	1.02
Pb	2008		98.92	1.08	0.00	0.00		100.00	0.00	0.00	0.00
	2017		95.56	2.05	1.37	1.02		97.61	1.71	0.68	0.00
As	2008		100.00	0.00	0.00	0.00		100.00	0.00	0.00	0.00
	2017		100.00	0.00	0.00	0.00		100.00	0.00	0.00	0.00
Cr	2008		99.46	0.54	0.00	0.00		100.00	0.00	0.00	0.00
	2017		100.00	0.00	0.00	0.00		100.00	0.00	0.00	0.00
Hg	2008		98.92	1.08	0.00	0.00		78.49	14.52	5.91	1.08
	2017		99.32	0.68	0.00	0.00		91.13	6.83	1.37	0.68
	2008	P_N_	98.92	1.08	0.00	0.00	RI	54.84	39.25	5.38	0.54
	2017		95.22	3.75	1.02	0.00		0.34	29.35	62.12	8.19

The results showed that in both periods the soil As and Cr $E_{r}^{i}$ values were less than 30 (Equations 3–5), which belonged to the slight ecological risk class, and the proportion of points with soil Hg at moderate and strong ecological risk decreased in 2017, with the proportion of points at medium ecological risk reduced from 14.52 to 6.83%, the proportion of points at strong ecological risk class reduced from 5.91 to 1.37%, and $E_{r}^{i}$ The proportion of sites with values greater than 120 belonging to very strong ecological risk decreased from 1.08 to 0.68%. The remaining two heavy metals Pb and Cd showed an increase in ecological risk after accumulation on the time scale of the last 10 years, with 0.68% (at strong ecological risk) and 1.02% (at very strong ecological risk) of sites in 2017, respectively. Through the RI values, the overall ecological risk of heavy metals in agricultural soils in the study area is on the rise, with the largest increase being in the strong ecological risk level, with the proportion of points rising from 5.38% in 2008 to 62.12% in 2017, indicating that the ecological risk of the agricultural land environment in the research region is becoming more serious with the development of urban construction should be taken seriously.

### 3.3 Heavy metal human health risk evaluation

Based on the 5 elements are recognized as having a serious risk to human health. The risks posed by these five elements to adults and children through different modes of intake can be known from Table 4 (Equations 6–12). We can see that the HQs of the five heavy metals in 2008 were ingestion > inhalation > dermal for either child and adult, showing that the ingestion route was the greatest risk route of exposure for the entire research region in 2008, and this is relatively in accordance with relevant research (22); while the magnitude of the HQ values and HI values were changed from adult > child > adult to child > adult, indicating that the non-carcinogenic risk of children is higher than that of adults at this stage. Therefore, with the development of urban construction should pay more emphasis on the protection of urban air quality and the cultivation of good living habits of children, which can effectively reduce the risk of inhalation pathway.

**Table 4:** Health risk evaluation results.

			Non-carcinogenic risk-				carcinogenic risk			
			HQ_ing_	HQ_inh_	HQ_dermal_	HI	CR_ing_	CR_inh_	CR_dermal_	TCR
Cd	2008	Children	6.87E-05	1.34E-04	8.62E-05	2.89E-04	-	1.219E-09	-	1.22E-09
		Adult	1.23E-05	1.26E-04	3.91E-06	1.42E-04	-	1.145E-09	-	1.14E-09
	2017	Children	1.86E-04	3.65E-04	2.34E-04	7.85E-04	-	3.305E-09	-	3.30E-09
		Adult	3.32E-05	3.42E-04	1.06E-05	3.86E-04	-	3.104E-09	-	3.10E-09
Pb	2008	Children	1.60E-02	-	1.53E-04	1.61E-02	-	-	-	-
		Adult	2.85E-03	-	6.96E-06	2.85E-03	-	-	-	-
	2017	Children	2.52E-02	-	2.42E-04	2.55E-02	-	-	-	-
		Adult	4.50E-03	-	1.10E-05	4.52E-03	-	-	-	-
As	2008	Children	2.56E-03	9.53E-04	5.73E-05	3.57E-03	1.71E-03	1.91E-07	1.15E-08	1.71E-03
		Adult	4.57E-04	8.95E-04	2.60E-06	1.35E-03	3.04E-04	1.79E-07	5.21E-10	3.05E-04
	2017	Children	5.90E-03	2.20E-03	1.32E-04	8.23E-03	3.93E-03	4.40E-07	2.64E-08	3.93E-03
		Adult	1.05E-03	2.07E-03	6.00E-06	3.12E-03	7.02E-04	4.13E-07	1.20E-09	7.03E-04
Cr	2008	Children	1.60E-02	6.24E-02	7.34E-06	7.84E-02	-	3.57E-06	2.86E-07	3.86E-06
		Adult	2.85E-03	5.86E-02	3.33E-07	6.15E-02	-	3.35E-06	1.30E-08	3.37E-06
	2017	Children	5.69E-02	2.22E-01	2.61E-05	2.79E-01	-	1.27E-05	1.02E-06	1.37E-05
		Adult	1.01E-02	2.09E-01	1.19E-06	2.19E-01	-	1.19E-05	4.63E-08	1.20E-05
Hg	2008	Children	3.12E-05	3.98E-05	2.18E-06	7.31E-05	-	-	-	-
		Adult	5.56E-06	3.74E-05	9.90E-08	4.30E-05	-	-	-	-
	2017	Children	3.71E-05	4.73E-05	2.59E-06	8.70E-05	-	-	-	-
		Adult	6.61E-06	4.45E-05	1.18E-07	5.12E-05	-	-	-	-
Sum	2008	Children	3.47E-02	6.35E-02	3.06E-04	9.84E-02	1.71E-03	1.92E-07	1.15E-08	1.71E-03
		Adult	6.17E-03	5.97E-02	1.39E-05	6.59E-02	3.04E-04	3.53E-06	1.35E-08	3.08E-04
	2017	Children	8.82E-02	2.25E-01	6.37E-04	3.14E-01	3.93E-03	1.31E-05	1.05E-06	3.94E-03
		Adult	1.57E-02	2.11E-01	2.89E-05	2.27E-01	7.02E-04	1.23E-05	4.75E-08	7.15E-04

“-” means no data available.

Based on the calculated carcinogenic risk values, it is observed that the CR values of Cd and Cr for different pathways for adults and children exhibit intake > inhalation > skin in both periods. Furthermore, adult TCR values for the two periods were 3.08 × 10^−4^ and 7.15 × 10^−4^ for both periods, between 1 × 10^−4^ and 1 × 10^−6^, respectively, which are acceptable risks. However, the TCR values for children were 1.71 × 10^−3^ and 3.94 × 10^−3^, both of which exceeded the upper limit of carcinogenic risk level. By further analysis of Cd, Cr and As, the contribution of Cd and Cr to the TCR values of children was less than or within the acceptable level and could be considered as no health hazard, while the contribution of As to the TCR values by the oral intake route in both periods exceeded 90%, indicating that the ingested heavy metal As was the primary contributor to the risk of cancer.

### 3.4 Analysis of heavy metal sources

#### 3.4.1 Correlation analysis

After nearly 10 years of rapid urbanization, a correlation analysis of the five heavy metals in the research region was conducted to find out whether the sources of the five heavy metals in arable soils are consistent and how they change. In case of a clear negative correlation among the elements, it means different sources of the elements or even some antagonistic effects (26). According to the data, Cd, Pb and Hg showed a two-by-two correlation in 2008, suggesting that they might share a common source, while Cr and As were remarkably negatively correlated, indicating that their sources in arable soils are different, and none of the other heavy metals of As were significantly correlated, so As may have a single source; while Cd and Pb were not significantly correlated in 2017, Pb and Cr were significantly positively The correlation coefficient is greater than 0.4, so it may have the same source. As and Hg have changed from no significant correlation with each element in 2008 to significant positive correlation. Cd has no significant correlation with other elements, so it may have a single source. This indicates that after the rapid urbanization process in the last decade, the sources of heavy metals in agricultural soils have been changed by human activities.

#### 3.4.2 Factor analysis

The data on soil heavy metal content in the research region for two periods were subjected to KMO test and Bartlett′s sphere test, with KMO test coefficients of 0.580 and 0.523 > 0.5, respectively, and Bartlett′s sphere test *p* < 0.05, indicating that each heavy metal element is highly correlated and suitable for factor analysis. The outcome (Table 5) revealed that the eigenvalues of both factors before 2008 were greater than 1, but the cumulative total explained variance was only 53.81%, which was not enough to explain significant portions of the information in the raw data, and after extracting by 3 factors, the cumulative total explained variance reached 71.95% > 70%, indicating that these 3 factors could reflect most of the information of all the data; the cumulative total explained variance of the first 3 factors in 2017 The variance was 78.42% > 70%, so these 3 factors can reflect most of the information of all the data (Equations 13,14).

**Table 5:** Correlation analysis of heavy metals in agricultural soils in Chengdu.

Element	Year	Cd	Pb	As	Cr	Hg
Cd	2008	1.000				
Pb		0.278^**^	1.000			
As		−0.220^**^	−0.038	1.000		
Cr		0.201^**^	0.075	−0.093	1.000	
Hg		0.148^*^	0.236^**^	0.049	0.087	1.000
Cd	2017	1.000				
Pb		0.080	1.000			
As		−0.082	−0.087	1.000		
Cr		0.008	0.592**	−0.208**	1.000	
Hg		0.125^*^	−0.085	0.239**	−0.196**	1.000

*indicates correlation (0.01 < *p* < 0.05), **indicates significant correlation (*p* < 0.01).

From the results of the factor analysis in 2008, the contribution of factor 1 was 31.54%, in which Cd, Pb had a large loading. From the correlation analysis, it can be seen that there is a significant positive correlation between Cd and Pb; after investigation, numerous chemical plants are distributed in the northwest of the study area, which mainly produce metal protective coatings, and a large number of nonferrous metal smelting and rolling processing plants are distributed in the southeast. cd is widely used in various chemical industries and is also a major by-product of coating production, and the mining of minerals and nonferrous metal smelting are important sources of Pb one of them. Some studies have shown that these heavy metals can cause the enrichment of Cd and Pb in soil through the emission of waste gas, wastewater and sludge, through atmospheric deposition, surface runoff and solid waste dumping (27, 28). In summary, factor 1 represents the “industrial source.”

The contribution of factor 2 is 22.27%, and the heavy metals with larger loadings are As and Hg. According to Table 3, it can be seen that there is 1.33% proportion of points with mild Hg pollution in the study area. It was found that the southern part of the study area was distributed with the most key pollution sources and sewage treatment plants in the whole Chengdu city in 2008, so it was presumed that the Hg pollution of agricultural soil in the study area might be caused by long-term river sewage irrigation; some studies showed that As and Hg are important constituent elements of pesticides (29), and pesticides containing Hg or inorganic As were widely used in agriculture before they were banned, but due to the difficulty of degradation of heavy metals, they were in the soil The pesticides containing Hg or inorganic As were widely used in agriculture before they were banned. Therefore, factor 2 is classified as “agricultural source.”

The contribution of factor 3 was 18.15% and the only heavy metal with a large load was Cr. The average content of Cr in agricultural soils in 2008 was lower than the background value in the study area, and it is generally believed that heavy metals associated with soil parent material composition are often elements with a low level of contamination, and the contamination evaluation (Table 3) also pointed out that soil Cr was in a clean state at the detected sites. Numerous studies have shown that the content of Cr in soil is close to that in the parent material, related to the rock-forming composition and less influenced by anthropogenic factors (25). The high value area is located in the parent material type of river sediment and basalt, and related studies show that these two parent materials are conducive to the enrichment of heavy metal Cr in soil (30), so it can be considered that factor 3 represents the parent material of soil formation, i.e., “natural source.”

The factor analysis in 2017 resolved a total of three potential factors with a cumulative variance explained of 78.42%. Among them, the contribution of factor 1 was 35.16%, and Pb and Cr were the main loading heavy metals. In the source analysis in 2008, Cr was considered as a “natural source” related to soil-forming parent material, and the planning and construction of tourist areas will certainly accelerate the weathering of the parent material and changes in soil properties, according to the survey from the completion of the scenic area to 2017, the reception of tourists increased by 11% year-on-year, which greatly enhanced the regional vehicle circulation, and some studies have shown that Pb is a major indicator of traffic The main marker of emissions, because Pb as long as it comes from fuel combustion of transportation, car engines and tire friction (31), and they cumulate in agricultural soils by atmospheric deposition and adsorption of airborne dust (32). Therefore, factor 1 can be considered to represent “transportation sources.”

Factor 2 contributed 22.82%, with heavy metals with high loadings being As and Hg. In the correlation analysis, As and Hg were significantly and positively correlated, in which the average content of As increased but was in the state of no pollution, and the average content of Hg decreased and the ratio of pollution index and ecological risk index decreased. From correlation analysis and pollution evaluation, the pollution sources are the same as those analyzed in 2008, so factor 2 is considered to represent “agricultural sources.”

The contribution of factor 3 was 20.44%, soil Cd was the main load. The average content of Cd increased by 66.67%, the proportion of pollution index increased, and the potential risk appeared a very strong level of ecological risk, according to the results of the factor analysis in 2008, Cd in 2008 for industrial sources of pollution, related to the 2008 pollution industry, which may be related to the rapid urbanization phase of the study area sub-circle development, the may be related to the rapid urbanization stage and the outward migration of industrial parks. Therefore, factor 3 represents “industrial source.”

After nearly 10 years of rapid urbanization, soil Pb pollution has changed from an “industrial source” to a “transportation source” and soil Cr from a “natural source” to a “transportation source.” “This indicates that human activities have significantly influenced the accumulation and distribution of heavy metals in agricultural soils, and have aggravated soil heavy metal pollution.

#### 3.4.3 APCS-MLR receptor model

For the determination of the contribution of each heavy metal source to the contamination stream, the APCS-MLR receptor model was used to model each heavy metal element in the research region. According to the fitted values obtained from the model compared with the measured values, a ratio close to 1 indicates a good fitting effect (33). The results in Table 6 show that the fitted/measured values for both periods are approaching 1. Furthermore, the complex correlation coefficients for all heavy metals are greater than 0.6. In summary, it can be seen that the model predictions can adequately represent the original information of the data.

**Table 6:** Soil heavy metal factor analysis component matrix.

Element	Year	Factor			Year	Factor		
		1	2	3		1	2	3
Cd	2008	0.744	−0.196	−0.133	2017	0.062	−0.001	0.932
Pb		0.648	0.355	−0.317		0.900	0.020	0.062
As		−0.361	0.726	0.339		−0.043	0.834	−0.250
Cr		0.476	−0.251	0.817		0.876	−0.165	−0.012
Hg		0.497	0.600	0.076		−0.129	0.722	0.364
Eigenvalue		1.577	1.113	0.907		1.758	1.141	1.022
Explaining the total variance/%		31.538	22.270	18.148		35.158	22.823	20.436
Cumulative explained total variance/%		31.538	53.807	71.955		35.158	57.981	78.417

**Figure 3:**
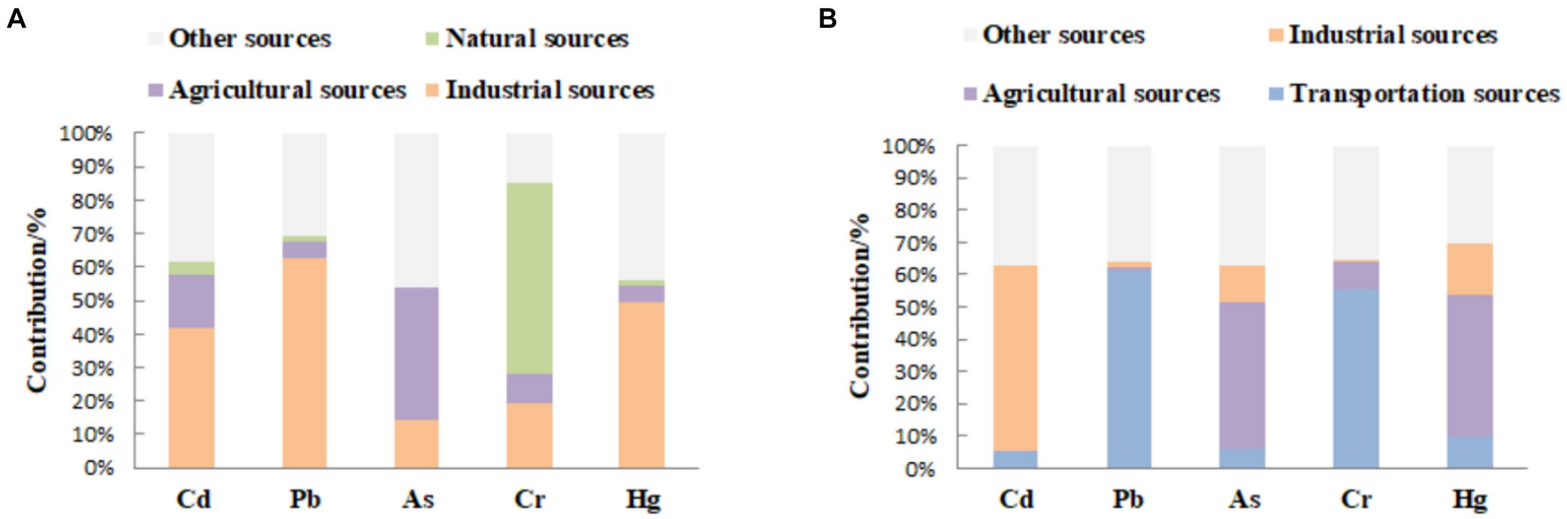
**(A)** Contribution of heavy metal pollution in the study area in 2008, **(B)** Contribution of heavy metal pollution in the study area in 2017.

Based on correlation analysis, factor analysis, geostatistical analysis and model analysis, the results of pollution source identification and quantitative source analysis were obtained (Figure 3).

The sources of heavy metals Cd, Pb and Hg in agricultural fields in Chengdu in 2008 were mainly industrial sources, and the contribution of industrial sources to these three was 41.97, 62.85 and 49.37%, respectively; meanwhile, agricultural sources also contributed more to Cd and As, 15.74 and 39.59%, respectively; followed by the contribution of natural sources to Cr up to 56.93%, and the contribution of the three pollution sources to The contribution of the three sources to its contribution rate is more consistent, indicating that the source of soil Cd pollution in the study area in 2008 is more complex. The white column (Figure 3) represents other pollution sources, whose contribution to Cr is as high as 58.10%.

Comparing with the quantitative source analysis results in 2017, Cr and Pb pollution increased after 10 years, the average content of soil Cd increased by 108.97%, the proportion of mild, moderate and severe pollution sample points of soil Pb pollution index increased from 1.08, 0 and 0% in 2008 to 2.05, 1.17 and 1.02%, transportation sources replaced natural sources and industrial sources. The contribution of agricultural sources to Cd decreased, but also caused the pollution of As and Hg, with the contribution of 45.63 and 44.01% respectively; the contribution of industrial sources to Cd increased, with the contribution of 57.33% (Table 7).

**Table 7:** Fitting results and prediction ratio of APCS-MLR model.

Element	Years	Multi-correlation coefficient	Fitting value/measured value
Cd	2008	0.609	1
	2017	0.870	0.999
Pb	2008	0.646	1
	2017	0.823	1
As	2008	0.772	1
	2017	0.751	1
Cr	2008	0.958	1
	2017	0.796	1
Hg	2008	0.612	0.999
	2017	0.681	0.999

The results of quantitative source analysis in the two periods show that the pollution sources of the five soil heavy metals have changed significantly due to the change of land use types and the transformation of soil properties during urbanization construction, and the contribution of the sources of the five heavy metals to their pollution levels has increased significantly, and the contribution of the unresolved sources to heavy metal pollution has changed from the dominance of individual heavy metals to the collective contribution of the five heavy metals, and their specific represented sources need to be further studied, heavy metal levels in cropland are an aggregate of multiple sources, subject to greater anthropogenic influence, the more extensive their sources. This is in accordance to the findings of many related research (34, 35).

## 4 Conclusion

The most rapidly developing time period of Chengdu urbanization is 2008 ~ 2017, this study combines the characteristics of Chengdu urbanization development (3-circle development), by collecting farmland soil samples in these two time points by circles, the results found that rapid urbanization contributes to the accumulation of soil Cd, As, and Cr and increases the proportion and level of soil contamination. At the same time the pathway of endangering human health risk shifted from ingestion to inhalation pathway, which is consistent with the reported decrease in air quality in the study area. Accelerated urbanization will change the shift in soil Cr sources, and transportation sources will replace natural sources as the main source of soil Cr accumulation, while increasing the contribution of soil Cd from industrial sources.

In conclusion, the results of this study can provide a theoretical basis for the prevention and remediation of heavy metal pollution in farmland soils in urbanized areas as well as for urban development planning and construction.

## Data availability statement

The datasets presented in this article are not readily available because data related to the study is not stored in a publicly accessible repository. The data that has been used is confidential. Requests to access the datasets should be directed to CH, 2021220013@sstu.icau.edu.cn.

## Author contributions

CH: Writing – original draft, Writing – review & editing. ZG: Software, Writing – original draft, Data curation. XM: Conceptualization, Investigation, Writing – review & editing. GL: Investigation, Project administration, Writing – review & editing. OD: Methodology, Validation, Writing – review & editing. YY: Resources, Supervision, Writing – review & editing.

## References

[1] Mandal A Voutchkov M. Heavymetal pollution of agricultural soils in central regions of KOREA. Int J Geosci. (1998) 39:698–704. doi: 10.1007/s002540050484

[2] Guan Q Wang F Xu C Pan N Lin J Zhao R. Source apportionment of heavy metals in agricultural soil based on PMF: a case study in Hexi corridor, Northwest China. Chemosphere. (2018) 193:189–97. doi: 10.1016/j.chemosphere.2017.10.151 29131977

[3] Ding Q Cheng G Wang Y Zhuang D. Effects of natural factors on the spatial distribution of heavy metals in soils surrounding mining regions. Sci Total Environ. (2017) 578:577–85. doi: 10.1016/j.scitotenv.2016.11.001 27839763

[4] Fei JC Min XB Wang ZX Pang ZH Liang YJ Ke Y. Health and ecological risk assessment of heavy metals pollution in an antimony mining region: a case study from South China. Environ Sci Pollut Res Int. (2017) 24:27573–86. doi: 10.1007/s11356-017-0310-x 28980103

[5] Yi Y Yang Z Zhang S. Ecological risk assessment of heavy metals in sediment and human health risk assessment of heavy metals in fishes in the middle and lower reaches of the Yangtze River basin. Environ Pollut. (2011) 159:2575–85. doi: 10.1016/j.envpol.2011.06.011 21752504

[6] Fei X Xiao R Christakos G Langousis A Ren Z Tian Y. Comprehensive assessment and source apportionment of heavy metals in Shanghai agricultural soils with different fertility levels. Ecol Indic. (2019) 106:105508. doi: 10.1016/j.ecolind.2019.105508

[7] Kong M Zhong H Wu Y Liu G Xu Y Wang G. Developing and validating intrinsic groundwater vulnerability maps in regions with limited data: a case study from Datong City in China using DRASTIC and Nemerow pollution indices. Environ Earth Sci. (2019) 78:262. doi: 10.1007/s12665-019-8255-7

[8] Sun W Yu J Xu X Zhang W Liu R Pan J. Distribution and sources of heavy metals in the sediment of Xiangshan Bay. Acta Oceanol Sin. (2014) 33:101–7. doi: 10.1007/s13131-014-0456-z

[9] Min X-B Xie X-D Chai L-Y Liang Y-J Li M Ke Y. Environmental availability and ecological risk assessment of heavy metals in zinc leaching residue. Trans Nonferrous Metals Soc China. (2013) 23:208–18. doi: 10.1016/s1003-6326(13)62448-6

[10] Facchinelli A Sacchi E Mallen L. Multivariate statistical and GIS-based approach to identify heavy metal sources in soils. Environ Pollut. (2001) 114:313–24. doi: 10.1016/S0269-7491(00)00243-8, PMID: 11584630

[11] Pekey H Karakas D Bakoglu M. Source apportionment of trace metals in surface waters of a polluted stream using multivariate statistical analyses. Mar Pollut Bull. (2004) 49:809–18. doi: 10.1016/j.marpolbul.2004.06.029 15530525

[12] Lang YH Li GL Wang XM Peng P. Combination of Unmix and PMF receptor model to apportion the potential sources and contributions of PAHs in wetland soils from Jiaozhou Bay, China. Mar Pollut Bull. (2015) 90:129–34. doi: 10.1016/j.marpolbul.2014.11.009 25434785

[13] Liu Y Liu G Yousaf B Zhang J Zhou L. Carbon fractionation and stable carbon isotopic fingerprint of road dusts near coal power plant with emphases on coal-related source apportionment. Ecotoxicol Environ Saf. (2020) 202:110888. doi: 10.1016/j.ecoenv.2020.110888 32585485

[14] Wu J Li J Teng Y Chen H Wang Y. A partition computing-based positive matrix factorization (PC-PMF) approach for the source apportionment of agricultural soil heavy metal contents and associated health risks. J Hazard Mater. (2020) 388:121766. doi: 10.1016/j.jhazmat.2019.121766 31818669

[15] Wang F. Yu H. Wang Z. Liang W. Shi G. Gao J., . . . Feng Y. (2021). Review of online source apportionment research based on observation for ambient particulate matter. Sci Total Environ, 762:144095. doi: 10.1016/j.scitotenv.2020.144095 33360453

[16] Bing L Chang-quan W Ting T Huan-xiu L Juan Y Quan LQ-I. Regional distribution and pollution evaluation of heavy metal pollution in TOPSOILS of the Chengdu PLAIN. J Nuclear Agricul Sci. (2009) 23:308–15. doi: 10.11869/hnxb.2009.02.0308

[17] Pang S Li T-X Zhang X-F Wang Y-D Yu H-Y. Spatial variability of cropland lead and its influencing factors: a case study in Shuangliu county, Sichuan province, China. Geoderma. (2011) 162:223–30. doi: 10.1016/j.geoderma.2011.01.002

[18] Qin B. City profile: Chengdu. Cities. (2015) 43:18–27. doi: 10.1016/j.cities.2014.11.006

[19] Cheng J-L Shi Z Zhu Y-W. Assessment and mapping of environmental quality in agricultural soils of Zhejiang Province, China. J Environ Sci. (2007) 19:50–4. doi: 10.1016/s1001-0742(07)60008-4, PMID: 17913153

[20] Hakanson L. An ecological risk index for aquatic pollution. Water Res. (1980) 14:975–1001. doi: 10.1016/0043-1354(80)90143-8

[21] Men C Liu R Xu F Wang Q Guo L Shen Z. Pollution characteristics, risk assessment, and source apportionment of heavy metals in road dust in Beijing, China. Sci Total Environ. (2018) 612:138–47. doi: 10.1016/j.scitotenv.2017.08.123 28850834

[22] Jiang Y Chao S Liu J Yang Y Chen Y Zhang A. Source apportionment and health risk assessment of heavy metals in soil for a township in Jiangsu Province, China. Chemosphere. (2017) 168:1658–68. doi: 10.1016/j.chemosphere.2016.11.088 27932041

[23] Totolo O Mosweu S. Spatial variability of selected soil properties in relation to different land uses in northern Kgalagadi (Matsheng). Botswana Int J Geosci. (2012) 03:659–63. doi: 10.4236/ijg.2012.34066

[24] Zhou F Huang GH Guo H Zhang W Hao Z. Spatio-temporal patterns and source apportionment of coastal water pollution in eastern Hong Kong. Water Res. (2007) 41:3429–39. doi: 10.1016/j.watres.2007.04.022 17572471

[25] Cheng G Wang M Chen Y Gao W. Source apportionment of water pollutants in the upstream of Yangtze River using APCS-MLR. Environ Geochem Health. (2020) 42:3795–810. doi: 10.1007/s10653-020-00641-z 32594417

[26] Dong LK Fang B. Analysis of spatial heterogeneity of soil heavy metals in tea plantations at the township scale - an example of high-quality tea plantations in Jiangsu and Zhejiang. Geogr Res. (2017) 36:391–404. doi: 10.11821/dlyj201702016

[27] Huang Y. Zhang S. Chen Y. Wang L. Long Z. Hughes S. S., . . . Liu C. (2020). Tracing Pb and possible correlated cd contamination in soils by using Lead isotopic compositions. J Hazard Mater, 385:121528. doi: 10.1016/j.jhazmat.2019.121528 31735468

[28] Kumar M. Furumai H. Kurisu F. Kasuga I., Tracing source and distribution of heavy metals in road dust, soil and soakaway sediment through speciation and isotopic fingerprinting. Geoderma. (2013) 211-212:8–17. doi: 10.1016/j.geoderma.2013.07.004

[29] Giersz J Bartosiak M Jankowski K. Sensitive determination of hg together with Mn, Fe, cu by combined photochemical vapor generation and pneumatic nebulization in the programmable temperature spray chamber and inductively coupled plasma optical emission spectrometry. Talanta. (2017) 167:279–85. doi: 10.1016/j.talanta.2017.02.018 28340721

[30] Sun S. S. Ao M. Geng K. R. Chen J. Q. Deng T. H. Li J. J., . . . Qiu R. L. (2022). Enrichment and speciation of chromium during basalt weathering: insights from variably weathered profiles in the Leizhou peninsula, South China. Sci Total Environ, 822:153304. doi: 10.1016/j.scitotenv.2022.153304 35090923

[31] Yang Z Lu W Long Y Bao X Yang Q. Assessment of heavy metals contamination in urban topsoil from Changchun City China. J Geochem Explor. (2011) 108:27–38. doi: 10.1016/j.gexplo.2010.09.006

[32] Pardyjak ER Speckart SO Yin F Veranth JM. Near source deposition of vehicle generated fugitive dust on vegetation and buildings: model development and theory. Atmos Environ. (2008) 42:6442–52. doi: 10.1016/j.atmosenv.2008.04.024

[33] Hingorani R Jimenez-Relinque E Grande M Castillo A Nevshupa R Castellote M. From analysis to decision: revision of a multifactorial model for the in situ assessment of NOx abatement effectiveness of photocatalytic pavements. Chem Eng J. (2020) 402:126250. doi: 10.1016/j.cej.2020.126250

[34] Duan Y Zhang Y Li S Fang Q Miao F Lin Q. An integrated method of health risk assessment based on spatial interpolation and source apportionment. J Clean Prod. (2020) 276:123218. doi: 10.1016/j.jclepro.2020.123218

[35] Hu Y Cheng H. Application of stochastic models in identification and apportionment of heavy metal pollution sources in the surface soils of a large-scale region. Environ Sci Technol. (2013) 47:3752–60. doi: 10.1021/es304310k 23496004

